# Does rest breed rust? An examination of DNP-Rest decisions and performance in the National Basketball Association regular and post-season

**DOI:** 10.3389/fspor.2022.977692

**Published:** 2022-10-18

**Authors:** Sean Pradhan, Travis J. Miller

**Affiliations:** ^1^School of Business, Menlo College, Atherton, CA, United States; ^2^Center for Sports Management, Menlo College, Atherton, CA, United States; ^3^Department of Psychology, Menlo College, Atherton, CA, United States

**Keywords:** professional basketball, rest, performance, National Basketball Association (NBA), post-season

## Abstract

Within the National Basketball Association (NBA), players and teams maintain that having healthy players sit out some games during the regular season may help them be more productive during the post-season. This decision to not play in order to rest the player, aptly noted as a *DNP-Rest* decision on injury reports, is in line with team and player goals, and fits with a growing body of evidence in support of the power of rest for health and performance. However, these practices conflict with some goals of the league, which has a vested interest in having the top talent play to attract broadcasters, advertisers, live spectators, and thus, enhance viewership. The current study is among the first to test the theory that strategically resting healthy players during the regular season results in better performance, as indicated by Player Efficiency Rating (PER) and Win Shares, during the post-season. Utilizing data from the 2016–17 through the 2020–21 NBA seasons, there was not sufficient evidence to suggest that resting more games during the regular season results in better performance in the post-season. Findings from a nested case-control study of 184 players (92 cases; 92 controls) also showed no differences in the change in performance from regular to post-season between cases of players who received rest during the regular season and matched controls. Although the restorative effects of rest might be considerable in the short term, the current study provides additional evidence to suggest that the impact may not carry over into the post-season.

## Introduction

“*We all need more rest […] we all wish we could have more rest.”*

– LeBron James, Los Angeles Lakers (1)

The value and importance of rest in sports has been and continues to be studied from many different angles, which include: (1) the physical aspects of rest (2), (2) the role of rest in injury (3), (3) the benefits of sleep (4), (4) short rest periods during training and performance (5), and (5) the psychological benefits of rest for recovery, learning, and expert performance (6). In particular, the National Basketball Association (NBA) as a league, acknowledges the importance of rest, specifically regarding the impact of sleep, days between performances, and frequency of performances on injury (7). The goals of the players and teams are in some ways at odds with the NBA as a league on this issue. Although the players and teams meticulously plan strategies to be at their best during the post-season, the league is concerned with increasing revenue and entertaining fans. In recent years, teams began resting healthy players, citing “rest” under the team injury report for their decision not to play the athlete (i.e., listed as *DNP-Rest*) (8). This strategy has been explicitly employed to reduce the likelihood of injury and fatigue, also called *load management*, and has increased among the NBA's most elite players (9). The player's and team's intentions are that by resting games during the regular season, they can perform at their best during the post-season.

“*[We will have] a very put together plan by our staff throughout the season to have our guys peak in April.”*

– Daryl Morey, former Houston Rockets General Manager and current Philadelphia 76ers President of Basketball Operations (9)

These issues first came to a head during the 2012–13 NBA season when the San Antonio Spurs rested four of their top players (i.e., Tim Duncan, Manu Ginobili, Tony Parker, and Danny Green) for a nationally televised game against the Miami Heat (10). The Spurs and their head coach Gregg Popovich described the decision as in line with their goals of winning a championship (which coincidentally was won by the Heat over the Spurs that year) (10). The NBA responded by fining the Spurs $250,000 for doing a disservice to the league and the fans (11). The NBA's argument for doing so was that the Spurs played the Heat in Miami only once during the regular season. By opting to rest their top players, it deprived the fans of seeing the league's top talent both in-person and *via* broadcast. Additionally, the game was nationally televised, and the league fears that games without the top talent will attract fewer viewers, thereby impacting how much television partners are willing to pay to broadcast games in the future (7, 11).

The NBA has worked toward resolving these issues with the players and teams by including rest among the variables taken into consideration when creating the NBA schedule (7). Researchers specifically urge the NBA to consider the science of sleep and rest along with educating players and team staff about these factors (12). There are specific logistical and financial constraints that the NBA must consider when setting the schedule (e.g., number of games, length of the season, arena availability, travel time) that get in the way of the schedule optimizing rest (7).

### Purpose and hypotheses

Despite the research on the importance of rest for sport performance, and the interest and discussion on resting players in the NBA, very few investigations to date have empirically tested if these decisions have their intended positive consequences. Early evidence taken from only 54 players spanning the 2005–2015 NBA seasons found no difference in post-season health (games missed due to injury) and performance (using mostly box-score statistics) between players who missed more regular season games for non-injury reasons (not specifically listed as *DNP-Rest*) and those who missed fewer regular season games for non-injury reasons (13). The current project extends this work, looking at more recent seasons, and a different conceptualization of rest decisions to test the theory presented by players and teams: *Strategically resting healthy players during the regular season results in better performance in the post-season*. Thus, we hypothesized that resting more games during the regular season would result in greater performance increases during the post-season.

Specifically, we utilized player efficiency rating (PER) and win shares (WS) per 48 min as our primary performance indicators in this study. PER is described as a per-minute productivity metric based on common box-score statistics, such as field-goals, free-throws, three-point shots, blocks, steals, turnovers, personal fouls, among others (14). WS is a compilation of points produced, offensive possessions, defensive rating, and marginal offense and defense that serves to quantify a player's contribution to team wins across a full 48-min game (15). These two metrics have been noted and implemented as markers of player performance in a variety of previous studies (16–19). We predict that there will be a positive relationship between these variables such that increases in games rested will be associated with increases in PER and WS from regular season to the post-season (**H**_**1**_). We also expect to find that players who have rested during the regular season will experience a greater change in PER and WS from the regular season to the post-season compared to those who did not receive DNP-Rest decisions during the regular season (**H**_**2**_).

## Methods

Player performance metrics for both the regular season and post-season were obtained from the publicly-available database, Basketball-Reference (20). Data on rest decisions were collected from Spotrac (21), a sports database containing information about player contracts, salaries, transactions, and other statistics. We extracted all available rest decisions from this source, which currently range from the 2016–17 to 2021–22 NBA seasons. Data collection occurred prior to the 2021–22 NBA post-season, so data from the 2021–22 season were not included in the current study. Overall, a total of 359 DNP-Rest decisions for 131 different players (*M*_*age*_ = 29.61 years, *SD* = 3.88) were listed by Spotrac (21) during the sample period. On average, players included in our sample were rested for approximately 2 games (*M* = 1.99, *SD* = 2.08). Across the period, there was an upward trend in the number of games rested per season (see Table 1).

**Table 1:** Listed DNP-Rest decisions across sample period (2016–17 to 2020–21).

**Season**	**Games rested by player^a^**	**Cash earned while resting^a^**	**Listed DNP-Rest decisions^b^**	**Number of unique players receiving DNP-Rest decisions^b^**
2016–17	1.68 (1.28)	$250, 605.12 ($287, 845.79)	42	25
2017–18	1.08 (0.29)	$108, 359.75 ($78, 402.38)	13	12
2018–19	1.77 (1.30)	$187, 778.42 ($219, 803.67)	92	52
2019–20	1.93 (1.87)	$241, 758.63 ($420, 422.18)	58	30
2020–21	2.52 (2.92)	$411, 357.16 ($912, 720.09)	154	61

a Values expressed as M (SD).

b Values indicate frequency.

### Data analysis

All statistical analyses were performed in RStudio (22) (Version 2022.02.1). A series of Spearman and repeated-measures correlations (23) examining the relationships between the number of games rested and the change in PER and WS per 48 min between the regular season and post-season were conducted. Next, we performed a nested case-control study to match players that rested during the regular season with other similar players that did not receive listed DNP-Rest decisions. Nested case-control study designs are typically used in the fields of medicine and epidemiology (24, 25). This method allows researchers to examine a cohort of individuals who have been diagnosed with a specific condition (i.e., in this case, players who received DNP-Rest decisions) and compare them with a selected sample of individuals that are matched on specific characteristics (26).

In our study, players receiving rest during the sample period were matched with controls based on *team post-season status, age group* (19–23 years, 24–28 years, 29–33 years, 34+ years), *position* (frontcourt, backcourt), and *regular season PER category* (<0–9.0, 9.0–11.0, 11.0–13.0, 13.0–15.0, 15.0–16.50, 16.50–18.0, 18.0–20.0, 20.0–22.5, 22.5–25.0, 25.0–27.5, 27.5–30.0, 30.0–35.0, 35+) (27). In all, 184 players (92 cases; 92 controls) were selected based on these criteria (see Table 2 for descriptive statistics). These matched pairs were used to compare individual players who rested during the regular season to other similar peers who did not rest. Using a series of mixed-effects multiple linear regression models, we then compared the change in PER and WS per 48 min from the regular season to the post-season between the matched cases and controls. Across these analyses, we accounted for player age, percentage of regular season games played, and regular season usage rate (i.e., an estimate of team possessions utilized by a particular player when on the court) (15). For each model, we computed both marginal and conditional *R*^2^ values, which denote the variance explained by either solely the fixed effects or the entire model (i.e., fixed and random effects), respectively (28).

**Table 2:** Descriptive statistics for matched cases and controls.

**Outcome**	**Cases (*n* = 92)**	**Controls (*n* = 92)**
Age group^a^	29–33	29–33
Age^b^	29.61 (3.83)	29.38 (3.95)
Percentage of games played (regular season)^b^	74.98 (18.32)	76.59 (21.42)
Usage percentage (regular season)^b^	22.17 (6.66)	21.32 (6.34)
Games played (post season)	11.04 (5.69)	8.07 (5.04)
Regular season PER category^a^	11–13	11–13
Change in PER (regular season – post-season)^b^	−1.79 (4.34)	−2.62 (6.46)
Regular season win shares per 48 min	0.14 (0.06)	0.14 (0.06)
Change in win shares per 48 min (regular season – post-season)^b^	−0.04 (0.07)	−0.06 (0.10)

a Values indicate mode category.

b Values expressed as M (SD).

## Results

### Correlations

The Spearman correlations revealed no meaningful relationship between the number of games rested and change in PER, ρ = 0.08, *p* = 0.46, 95% CI: [−0.15, 0.31], or change in WS per 48 min, ρ = 0.08, *p* = 0.46, 95% CI: [−0.16, 0.31]. Given that 40 players received multiple DNP-Rest decisions during the sample period, we conducted follow-up repeated-measures correlations to investigate the effects of the number of games rested on these two metrics to account for individual player variance. Results were consistent with the Spearman correlations, indicating no significant correlations between games rested and the change in PER, *r*_*rm*_ = −0.36, *p* = 0.10, 95% CI: [−0.69, 0.10], or WS per 48 min, *r*_*rm*_ = −0.01, *p* = 0.97, 95% CI: [−0.43, 0.45].

### Case-control comparisons

The mixed-effects models revealed no significant differences between players that rested during the regular season and matched controls on changes in PER or WS per 48 min (*p*-values > 0.05). Overall, the models explained a small proportion of the variance according to the $R_{Marginal}^{2}$ values (<0.10). Table 3 provides the full results of the comparisons from the mixed-effects models.

**Table 3:** Linear mixed-effects model results for change in player efficiency rating (PER) and win shares (WS) per 48 min.

	***Change in PER*** ***(**$R_{Marginal}^{2}$ = **0.07;**$R_{Conditional}^{2}$ = **0.33)***			***Change in WS per 48 min*** ***(**$R_{Marginal}^{2}$ = **0.01; **$R_{Conditional}^{2}$ = **0.07)***		
**Predictor**	***β (SE)***	***95% CI***	***p***	***β (SE)***	***95% CI***	***p***
Rest	0.07 (0.07)	−0.08, 0.21	0.38	0.08 (0.08)	−0.07, 0.23	0.28
**Covariates**
Position	−0.03 (0.14)	−0.30, 0.24	0.83	−0.08 (0.14)	−0.36, 0.20	0.59
Percentage of games played (regular season)	−0.03 (0.08)	−0.18, 0.12	0.67	−0.04 (0.08)	−0.19, 0.12	0.64
Usage percentage (regular season)	−0.06 (0.09)	−0.23, 0.12	0.52	−0.002 (0.08)	−0.16, 0.16	0.98
Age group (24–28)^a^	0.33 (0.32)	−0.29, 0.95	0.30	0.05 (0.37)	−0.67, 0.78	0.88
Age group (29–33)^a^	0.28 (0.31)	−0.33, 0.89	0.36	0.13 (0.36)	−0.58, 0.84	0.71
Age group (34–40)^a^	0.28 (0.33)	−0.38, 0.93	0.41	0.17 (0.39)	−0.59, 0.94	0.65

Standardized coefficients reported.

a Reference level = age group (19–23).

## Discussion

To our knowledge, the present investigation offers the second quantitative study into the impact of player rest on performance in professional sports (13). Findings from the current study revealed no association between the number of games rested and validated measures of performance (i.e., PER and WS per 48 min) among NBA players. Due to the limited number of DNP-Rest decisions during the sample period, we conducted a nested case-control study to match cases of players who rested during the regular season with similar controls relative to team post-season status, age group, position, and regular season PER category. Analyses of these data did not reveal any differences in changes in PER and WS per 48 min between the regular and post-seasons among the cases and matched controls.

Thus, it appears that the restorative effects of rest during the regular season may not carry over into the post-season, at least with respect to the performance indicators examined in our study. Our findings are consistent with research by Belk et al. (13), which also investigated other metrics including box-score statistics, such as points per game, assists per game, true shooting percentage, blocks, steals, and number of post-season games missed because of injury. We provide both updated evidence to support Belk et al., and also novel findings with respect to the lack of specific differences in performance between the regular season and post-season as indicated by the change scores for PER and WS per 48 min.

Despite the evidence supporting the various avenues by which rest is helpful for health and performance (2–6), and the testimonials from players and teams that it is (1, 8–10), we did not find support for the theory that intentionally resting players for the regular season games leads to better performance in the post-season. Although these issues are admittedly more complicated than a single study can address, this evidence may suggest that the changes that the NBA has made in recent years to consider rest in the schedule making process (e.g., reducing the number of times teams play games 2 days in a row or four games in five nights) is sufficient to ensure players are able to perform consistently in the post-season. Or, at least that, resting players for a few games in addition to these schedule changes does not improve post-season performance to a measurable degree.

### Limitations and future directions for research

Several limitations are evident in the current study. First, our models had relatively low explanatory power according to the marginal and conditional *R*^2^ values. Consequently, there may be a variety of other factors related to the impact of rest that we did not consider, such as load management, injury mitigation, and competitive importance of impending games. In addition, the effects of DNP-Rest decisions may not simply be cumulative but could rather only have short-term benefits on player performance. Thus, future research should explore how decisions to rest players affect their performance in the next game. The scope of our investigation was also limited to only PER and WS per 48 min as markers of performance in our study.

Despite an absence of evidence that resting healthy players during the regular season leads to improved post-season performance, this should not necessarily be taken as confirmation that there is no effect. The current study only took into account specific instances where rest was the stated reason a player missed a game. However, there are many other reasons, including the stigma and fines from the league associated with resting healthy players, why NBA teams might choose to rest healthy players but might not report it as a *DNP-Rest*. Alternatively, teams may choose to simply cite a minor or recent injury a player may have had as the primary reason for not playing in a game instead of rest (e.g., general soreness) (29). Consequently, future research should also quantify rest in other ways by using minutes played or distance traveled, use qualitative evidence to explore the psychological effects of rest, and investigate both team- and individual-level performance factors.

## Conclusion

The current study used evidence from the 2016–17 to 2020–21 NBA seasons to investigate the impact that resting healthy players during the regular season has on player performance in the post-season. Findings suggest that resting healthy players during the regular season does not translate to improved performance during the post-season. The current evidence is at odds with the beliefs of players and teams, whose experience and perspectives tell them that resting does indeed impact health and performance. At present, the evidence countering the player and team perspectives is not so definitive that their expertise should be ignored. We encourage future research to continue studying these practices to verify the impact of rest on player performance.

## Data availability statement

Data obtained from Basketball-Reference (https://www.basketball-reference.com/) are publicly-available. Data from Spotrac may be retrieved *via* a Premium Account (https://www.spotrac.com/register/).

## Author contributions

SP conceptualized the study, handled data collection and statistical analysis, and wrote the manuscript. TM conceptualized the study and wrote the manuscript. All authors contributed to the article and approved the submitted version.

## Conflict of interest

The authors declare that the research was conducted in the absence of any commercial or financial relationships that could be construed as a potential conflict of interest.

## Publisher's note

All claims expressed in this article are solely those of the authors and do not necessarily represent those of their affiliated organizations, or those of the publisher, the editors and the reviewers. Any product that may be evaluated in this article, or claim that may be made by its manufacturer, is not guaranteed or endorsed by the publisher.
